# Characteristics of peripheral refractive errors in eyes of patients with non-amblyopic myopic anisometropia

**DOI:** 10.1186/s12886-024-03527-1

**Published:** 2024-06-21

**Authors:** Gengmin Tong, Yuanhui Jin, Hongyan Wu, Yao Zhou

**Affiliations:** Dongyang people’s Hospital, Dongyang, 322100 China

**Keywords:** Multispectral refraction topography, Relative peripheral refractive errors, Anisometropia

## Abstract

**Background:**

This study aims to investigate relative peripheral refractive (RPR) characteristics in children with non-amblyopic myopic anisometropia and explore potential associations between relative peripheral refractive errors (RPRE) and myopia.

**Methods:**

Relative peripheral refractive errors were assessed in 64 children diagnosed with non-amblyopic myopic anisometropia utilizing multispectral refraction topography (MRT). Two eyes of each patient were divided into into the more myopia eyes group (ME) and the fellow eyes group (FE). Evaluated parameters encompassed total defocus values (TRDV), defocus values at eccentricities spanning 0 to 15 degrees (RDV-15), 0 to 30 degrees (RDV-30), 0 to 45 degrees (RDV-45), as well as superior (RDV-S), inferior (RDV-I), temporal (RDV-T), and nasal (RDV-N) positions.

**Results:**

The study revealed a noteworthy contrast in TRDV values between Group ME (0.52 ± 0.36) and Group FE (0.17 ± 0.41), with a substantial significance (P < 0.0001). While no significant RDV-15 difference emerged between Group ME (0.01 ± 0.05) and Group FE (-0.01 ± 0.07) (P > 0.05), a meaningful RDV-30 difference existed between Group ME (0.11 ± 0.14) and Group FE (0.03 ± 0.19) (P = 0.0017). A significant discrepancy in RDV-45 was also observed between Group ME (0.39 ± 0.29) and Group FE (0.13 ± 0.34) (P < 0.001). Notably, RDV-I and RDV-T positions demonstrated marked differences between Group ME and Group FE (P < 0.0001), whereas no significant disparity was noted in RDV-S and RDV-N positions (P > 0.05).

**Conclusion:**

Eyes exhibiting greater myopia manifested more hyperopic peripheral defocus in the context of anisometropia. MRT as a novel ophthalmic evaluation technique, holds promising potential for broader clinical applications in the future.

## Background

Myopia, among the most prevalent eye diseases globally, is projected to affect 50% of the population by 2050 without effective intervention measures [1]. The prevalence of myopia varies with ethnicity and is particularly high among individuals of East Asian descent [2, 3]. Higher levels of myopia are associated with an increased risk of sight-threatening complications. While the exact pathogenesis of myopia remains unclear, recent research has emphasized the significance of peripheral hyperopic defocus. Studies suggest that relative hyperopic defocus has a significant impact on axial length growth and myopia progression [4, 5].

Multispectral refractive topography (MRT) is an innovative device utilized for assessing relative peripheral refractive errors (RPRE), enabling quantification of retinal hyperopic defocus [6]. This technique offers a comprehensive assessment of ocular refractive status, encompassing myopia and anisometropia. MRT serves as a non-invasive, rapid, and precise tool for diagnosing and monitoring refractive errors [7].

Anisometropia refers to a refractive error distinguished by a substantial difference in the refractive power between the two eyes. Unlike traditional cohort studies comparing myopes with emmetropes, which are frequently affected by confounding variables like age, gender, and environment, anisometropia offers a distinctive experimental framework for investigating myopia progression [8]. This is due to the observation of asymmetric ocular growth in individuals with anisometropia, occurring even under identical genetic and environmental influences.

This study aimed to determine whether interocular asymmetries in RPRE at different retinal regions were evident in human eyes with myopic anisometropia. We measured RPRE at different retinal regions in patients with myopic anisometropia using MRT to explore associations between RPRE and myopia. Our objective is to contribute to the advancement of more effective strategies for myopia prevention and management.

## Data and methods

### Inclusion and exclusion criteria

All participants were of Chinese ethnicity and had non-amblyopic myopic anisometropia, characterized by a minimum interocular difference of 1.00 diopters (D) in spherical-equivalent (SE) refractive error. Both eyes of each participant had a best corrected visual acuity of 0.00 LogMAR or better. Participants with significant ocular diseases, strabismus, other visual dysfunctions, recent eye medication use within the last six months, systemic illnesses, or those wearing orthokeratology or other contact lenses were excluded from the study.

### Methods

This is a retrospective study. Sixty-four subjects underwent measurements using the non-contact optical biometer, automatic refractometer, subjective refraction, and MRT (MSI C2000, THONDAR, China) from May to September 2023 at the outpatient Department of Ophthalmology, Dongyang People’s Hospital. The MRT examination was conducted in a darkroom, and all patients had a pupil diameter greater than 5.2 mm. The measured parameters included total defocus values (TRDV), defocus values at eccentricities ranging from 0 to 15 (RDV-15), 30 (RDV-30), and45 (RDV-45) degrees, as well as defocus values at superior (RDV-S), inferior (RDV-I), temporal (RDV-T), and nasal (RDV-N) regions. The spherical equivalent (SE) was calculated using the formula DS + DC/2, where DS denotes the diopter sphere and DC denotes the diopter cylinder. The SE was used to categorize each patient’s two eyes into the more myopia eyes group (ME) and the fellow eyes group (FE).

### Statistical analysis

Continuous data were presented as mean with standard deviation along with the range of distribution. Categorical data were expressed by counts, fractions, or percentages. For responsive variables such as RDV were transformed for normal distribution if the data were skewed. Mean RDV values of different retinal regions were compared between the more myopic eyes and the fellow eyes by using paired t-tests. All tests were two-tailed unless otherwise noted. Type I error was set at 5% and *P* < 0.05 was considered statistically significant. Statistical analysis was performed using SAS JMP software (JMP 14 Pro).

## Results

The characteristics of the anisometropic subjects are summarized in Table 1. 64 healthy teenagers (mean age 11.89 ± 1.83 years, 33 males and 31 females) were enrolled in this study. 46 patients exhibited a higher degree of myopia in their right eye. There was a significant difference in the mean values of SE, DS, DC, AL, and K1 between the two groups.

**Table 1:** The characteristics of the anisometropic subjects

	Age(y)	Gender (F/M)	More myopic eye (*R*/L)	SE (D)	DS(D)	DC(D)	AL (mm)	K1	K2
ME	11.89 ± 1.83	31/33	46/18	-2.33 ± 0.82	-2.24 ± 0.83	-0.20 ± 0.27	24.85 ± 0.87	42.38 ± 1.34	43.36 ± 1.50
FE	11.89 ± 1.83	31/33	46/18	-0.37 ± 0.80	-0.25±-0.78	-0.27 ± 0.31	23.96 ± 0.77	42.23 ± 1.36	43.35 ± 1.52
P	-	-	-	< 0.001	< 0.001	0.0275	< 0.001	< 0.001	0.49
95%CI	-	-	-				0.7811, 0.10027	0.0748, 0.2322	-0.13859, 0.0757

SE. Spherical equivalent; DS. Diopter sphere; DC. Diopter cylinder; AL. Axial length; K. Keratometry; ME. More myopia eyes group; FE. Fellow eyes group

**Fig. 1:**
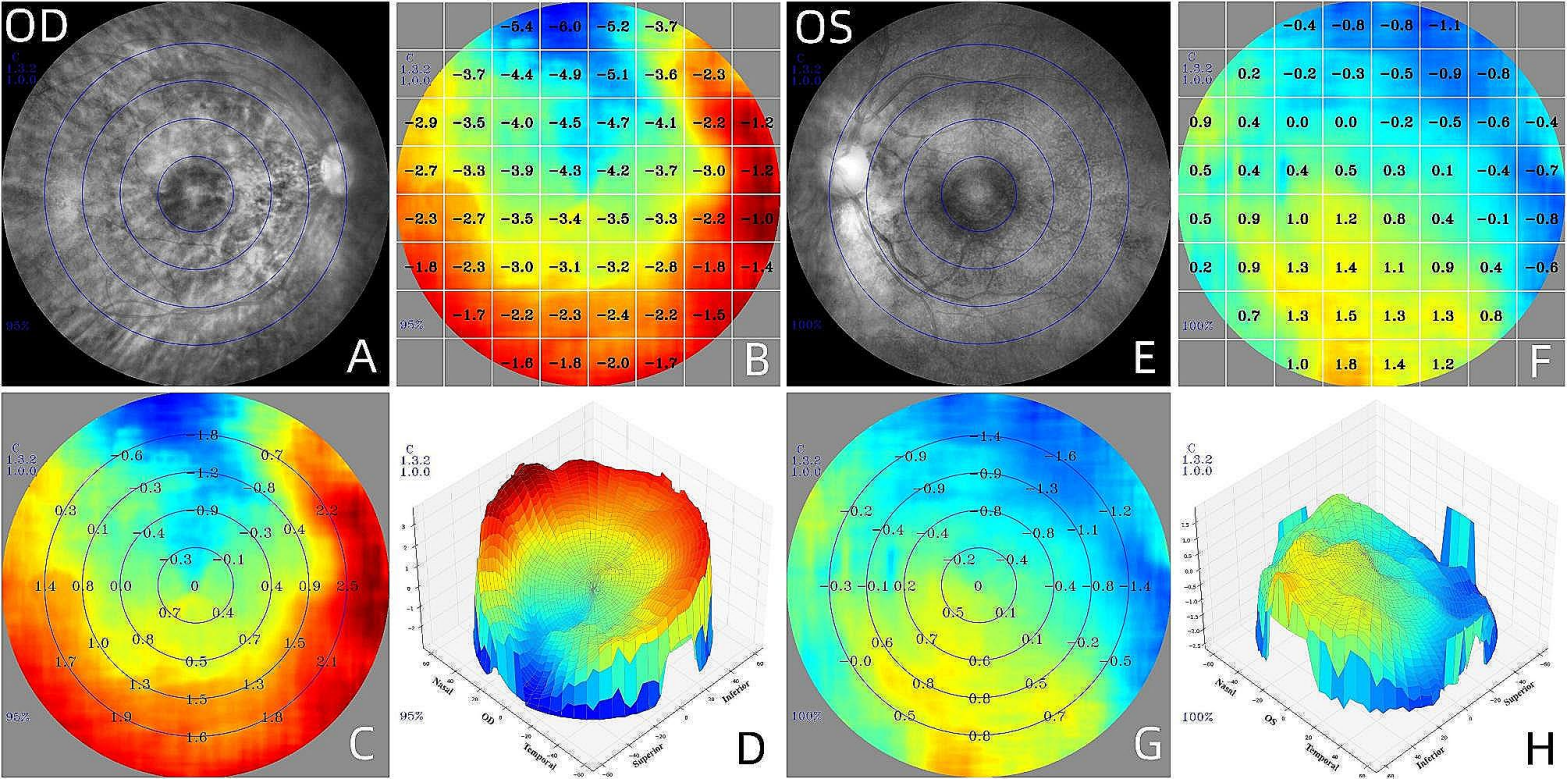
The RDV image data of a 10-year-old child diagnosed with anisometropia.(**A**, **E**). Fundus photograph of ranges to measure peripheral refraction; (**B**, **F**). The refraction of absolute refraction value, each block is expressed in diopters. (**C**, **G**). The relative peripheral refraction defocus value. The innermost circle stands for RDV-10; The second circle stands for RDV-20; The third circle stands for RDV-30; The fourth circle stands for RDV-40. (**D**, **H**). A direct view of the relative fraction status of the retinal by three-dimensional images viewing from nasal, temporal, superior and inferior

The RDV values of the two groups are shown in Table 2. There was a significant difference in the value of TRDV between Group ME (0.52 ± 0.36) and Group FE (0.17 ± 0.41), (*P* < 0.0001). In the RDV-15 range, there was no significant difference in the RDV-15 values between Group ME (0.01 ± 0.05) and Group FE (-0.01 ± 0.07), (*P* > 0.05). However, a significant difference was found in the RDV-30 values between Group ME (0.11 ± 0.14) and Group FE (0.03 ± 0.19), (*P* = 0.0017). In the range of RDV-45, we also can find a significant difference between Group ME (0.39 ± 0.29) and Group FE (0.13 ± 0.34), (*P* < 0.001). Significant differences were found between Group ME and Group FE in the RDV-I and RDV-T positions (*P* < 0.0001). No significant differences were found between Group ME and Group FE in the RDV-S and RDV-N positions (*P* > 0.05). Figure 1 presents the RDV image data of a 10-year-old child diagnosed with anisometropia, illustrating the noticeable variation in RDV between the patient’s bilateral eyes.

**Table 2:** The RDV values of the two groups

	TRDV	RDV-15	RDV-30	RDV-45	RDV-S	RDV-I	RDV-T	RDV-*N*
ME	0.52 ± 0.36	0.01 ± 0.05	0.11 ± 0.14	0.39 ± 0.29	0.01 ± 0.60	0.64 ± 0.50	0.66 ± 0.52	0.82 ± 0.79
FE	0.17 ± 0.41	-0.01 ± 0.07	0.03 ± 0.19	0.13 ± 0.34	-0.01 ± 0.80	0.20 ± 0.76	-0.08 ± 0.74	0.71 ± 0.80
95%CI	0.2349,0.4557	-0.0014,0.043	0.03384,0.13929	0.16955,0.3542	-0.0672,0.29599	0.27222,0.61403	0.57313,0.90281	-0.0852,0.30461
P	< 0.0001	0.0657	0.0017	< 0.001	0.2129	< 0.0001	< 0.0001	0.2651

TRDV. peripheral refractive error from center to peripheral 53°of retina; RDV-15. defocus values at eccentricities ranging from 0 to 15 of retina; RDV-30. defocus values at eccentricities ranging from 0 to 30 of retina; RDV-45. defocus values at eccentricities ranging from 0 to 45 of retina; RDV-S. refraction difference value-superior; RDV-I. refraction difference value-inferior; RDV-T. refraction difference value- refraction difference value-temporal; RDV-N. refraction difference value-nasal;

## Discussion

The prevalence of myopia is increasing globally, particularly in East Asia [2]. However, the underlying causes of this trend remain incompletely understood. Some theories propose that peripheral defocus significantly influences eye growth [4, 9]. Animal experiments have demonstrated that artificially induced hyperopic or myopic defocus can modify eye growth and refractive changes [4, 10, 11]. Mutti et al. [12] reported that children with myopia experience more relative hyperopic defocus than those with emmetropia, suggesting that hyperopic defocus may contribute to an elongation of the eye axis length. In patients with anisometropia, both eyes function in the same environment, but exhibit a significant difference in refractive power between them. Significant differences in TRVD were observed in the eyes of patients with anisometropia in this study (*P* < 0.05). Our study revealed that eyes with high myopia exhibit greater relative hyperopic peripheral defocus, low myopia exhibit less relative hyperopic peripheral defocus, while eyes with emmetropia and hyperopia tend to display relative myopic peripheral defocus. These findings are consistent with previous research on the topic [13]. To our knowledge, this is the first study to compare interocular differences using MRT in teenagers with anisometropia.

In this study, we investigated peripheral refraction at different retinal eccentricities and observed that Group ME displayed a statistically significant difference compared to Group FE in the RDV 30–45 range (*P* < 0.05). This result aligns with the findings of Lu et al.‘s [14] study, which reported an increase in myopia with an increase in RDV 30–45, indicating that peripheral refraction within the 30 to 45-degree range from the fovea may be more closely linked to myopia development.

David et al. [15] discovered that myopia has a greater impact on the peripheral refraction of adult eyes in the horizontal visual field compared to the vertical visual field. Zhao et al. [6] identified a noteworthy distinction between eyes with low myopia and those with moderate myopia in terms of relative peripheral refraction at positions RDV-N, RDV-S, and RDV-I. However, no significant variance was observed in the RDV-T position, diverging from our findings. In our investigation, significant disparities were detected between the two groups in RDV-T and RDV-I positions (*P* < 0.05), while no considerable distinctions manifested in other regions. These findings indicate a potential deviation from previous studies. Our interpretation speculates that peripheral refraction within the temporal and inferior regions may exert a pivotal influence on axial elongation. This assumption is based on the broader visual fields in these regions and the tendency of students with anisometropia to adopt tilted head positions while writing. Such postural adjustments could conceivably affect the imaging of the temporal and inferior sectors of the retina. However, further experiments are needed to validate these hypotheses.

The specific mechanism between peripheral hyperopia and axial growth remains controversial. Most viewpoints argue that peripheral hyperopia defocus leads to axial myopia progression, and a small number of views believe that peripheral hyperopic defocus is unrelated to the development of axial [16]. Several scholars have suggested that prolonged axial length may lead to peripheral hyperopia, implying a potential correlation between the two factors. Numerous studies have documented the ocular features of anisometropic amblyopia, indicating that the asymmetry in refractive errors is primarily axial in nature. Additionally, it seems that there is minimal involvement of the anterior segment in this condition [17, 18]. Our experimental findings support this notion, as we observed a positive correlation between eye axial length and the TRDV. Moreover, we observed that longer eye axial length is associated with increased peripheral hyperopia defocus within the RDV range of 30–45. This suggests that peripheral hyperopia defocus in this area may contribute to myopia development. Recently, progressive multifocal soft contact lenses, peripheral defocus glasses, and corneal reshaping lenses have gained popularity in ophthalmology. These methods can all delay the progression of myopia by reducing peripheral hyperopic defocus [19–22]. Li et al. [23] utilized MRT to measure the relative peripheral refraction of myopic children wearing orthokeratology lenses. They found that the RPR of children showed relative myopic defocus after wearing orthokeratology lenses. According to our research findings, it appears crucial to focus on retinal defocus status at the temporal and the 30 to 45 degrees distance from the fovea. We can utilize the results of MRT examinations to guide the treatment strategies for myopia and the personalized customization of lenses.

This study has some limitations that warrant acknowledgment. Firstly, the MRT employed in this study is a novel technique that has not been widely utilized. Therefore, its accuracy and repeatability need further verification through subsequent studies. Secondly, the influence of accommodation on MRT results was not accounted for in this investigation. Thirdly, owing to the cross-sectional design of this study, it was not feasible to track further progress over time. Finally, the sample size in this research was limited, and further studies with larger sample sizes are needed to validate the conclusions drawn in this study.

In conclusion, eyes with more myopia exhibit more hyperopic peripheral defocus in patients with anisometropia. As a novel ophthalmic examination technique, MRT may find broader applications in clinical practice in the future. It can enhance the diagnosis and prediction of myopia, as well as aid in selecting optimal treatment strategies.

## Data Availability

The datasets during and/or analyzed during the current study available from the corresponding author on reasonable request.
